# Supplementary material for: N-glycosylation in the protease domain of trypsin-like serine proteases mediates calnexin-assisted protein folding
Source: eLife. 2018 Jun 11;7:e35672. doi: 10.7554/eLife.35672 (PMC6021170; doi:10.7554/eLife.35672)
Supplement: Supplementary file 1. [file elife-35672-supp1.docx]

Supplementary File 1. Proteins identified in proteomic analysis

| protein | UniProt | spectral count | |
| --- | --- | --- | --- |
|  | accession | WT | N1022Q |
| 14-3-3 protein sigma | P31947 | 26 | 2 |
| 40S ribosomal protein S17 | A0A075B716 | 10 | 11 |
| 40S ribosomal protein S18 | P62269 | 9 | 17 |
| 40S ribosomal protein S3 | P23396 | 7 | 16 |
| 40S ribosomal protein S4, X isoform | P62701 | 9 | 13 |
| 40S ribosomal protein SA | A0A024R2P0 | 18 | 7 |
| Alpha-enolase | P06733 | 22 | 5 |
| Annexin A2 | P07355 | 57 | 31 |
| Arginase-1 | P05089 | 27 | 9 |
| ATP synthase subunit alpha, mitochondrial | P25705 | 30 | 21 |
| ATP synthase subunit beta, mitochondrial | P06576 | 17 | 16 |
| Beta actin variant (Fragment) | Q53G99 | 115 | 79 |
| Beta globin (Fragment) | B3VL05 | 10 | 18 |
| BiP | P11021 | 48 | 94 |
| Bleomycin hydrolase | Q13867 | 15 | 1 |
| Brain acid soluble protein 1 | P80723 | 12 | 9 |
| Calmodulin-like protein 5 | Q9NZT1 | 39 | 14 |
| Calnexin | P27824 | 13 | 27 |
| Calreticulin | P27797 | 17 | 15 |
| Caspase 14, apoptosis-related cysteine peptidase | B2CIS9 | 22 | 15 |
| Catalase | P04040 | 12 | 3 |
| Chromosome 7 open reading frame 24 | A0A090N7V5 | 15 | 3 |
| CKAP4 protein (Fragment) | Q6NWZ1 | 19 | 17 |
| Class IVb beta tubulin | Q8IWP6 | 21 | 4 |
| Cystatin-A | C9J0E4 | 13 | 4 |
| Delta-1-pyrroline-5-carboxylate synthase | P54886 | 6 | 23 |
| Dolichyl-diphosphooligosaccharide--protein glycosyltransferase subunit 1 | P04843 | 6 | 11 |
| Elongation factor 1-alpha 1 | P68104 | 29 | 12 |
| Elongation factor 2 | P13639 | 28 | 2 |
| Epididymis luminal protein 4 | D0PNI1 | 20 | 6 |
| Fatty acid-binding protein, epidermal | Q01469 | 45 | 9 |
| Filaggrin-2 | Q5D862 | 70 | 28 |
| Galectin-3-binding protein | B4DVE1 | 26 | 21 |
| Galectin-7 | P47929 | 27 | 0 |
| GCT-A5 light chain variable region (Fragment) | A0A0X9UWL5 | 150 | 195 |
| Glyceraldehyde-3-phosphate dehydrogenase | P04406 | 80 | 38 |
| Heat shock cognate 71 protein | E9PKE3 | 12 | 9 |
| Heat shock protein 70 1B | A0A0G2JIW1 | 33 | 13 |
| Heat shock protein 90 beta-1 | P14625 | 52 | 102 |
| Heat shock protein beta-1 | P04792 | 30 | 3 |
| Histone H2B type 1-J | P06899 | 11 | 1 |
| Histone H4 | P62805 | 27 | 9 |
| Hypoxia up-regulated protein 1 | Q9Y4L1 | 8 | 15 |
| Insulin-degrading enzyme | P14735 | 11 | 0 |
| Involucrin | B4DU44 | 11 | 0 |
| Isoform 2 of Heat shock protein HSP 90-alpha | P07900-2 | 18 | 15 |
| Isoform 2 of Serpin B12 | Q96P63-2 | 15 | 2 |
| Isoform 5 of Protein disulfide-isomerase A6 | Q15084-5 | 24 | 40 |
| L-lactate dehydrogenase A chain | P00338 | 34 | 8 |
| low density lipoprotein receptor-related protein associated protein 1 (LRPAP1), mRNA | B2R6S9 | 15 | 22 |
| Malate dehydrogenase, mitochondrial | P40926 | 25 | 28 |
| Mitochondrial heat shock 60kD protein 1 variant 1 | B3GQS7 | 29 | 32 |
| Neutral alpha-glucosidase AB | B4DJ30 | 19 | 43 |
| Peptidyl-prolyl cis-trans isomerase B | P23284 | 15 | 12 |
| Peroxiredoxin-1 | Q06830 | 34 | 17 |
| Peroxiredoxin-4 | Q13162 | 21 | 30 |
| Polyubiquitin-C (Fragment) | F5H2Z3 | 16 | 13 |
| Prelamin-A/C | P02545 | 58 | 4 |
| Probable Xaa-Pro aminopeptidase 3 | Q9NQH7 | 16 | 11 |
| Prohibitin-2 | F5GY37 | 16 | 12 |
| Protein disulfide-isomerase | P07237 | 20 | 22 |
| Protein disulfide-isomerase A3 | P30101 | 49 | 61 |
| Protein disulfide-isomerase A4 | P13667 | 39 | 65 |
| Protein kinase C substrate 80K-H, isoform CRA_a | A0A024R7F1 | 15 | 27 |
| Protein LOC100653049 | A0A140TA62 | 26 | 0 |
| Protein POF1B | Q8WVV4 | 21 | 3 |
| Protein S100 | B2R4M6 | 16 | 0 |
| Protein S100-A7 | P31151 | 26 | 19 |
| Protein S100-A8 | P05109 | 15 | 10 |
| Protein-glutamine gamma-glutamyltransferase E | Q08188 | 33 | 3 |
| Putative uncharacterized protein DKFZp666I134 (Fragment) | Q658S9 | 6 | 11 |
| Pyruvate kinase PKM | P14618 | 47 | 13 |
| Reticulocalbin-1 | Q15293 | 16 | 22 |
| Serpin B3 | P29508 | 19 | 2 |
| Serpin H1 | P50454 | 11 | 19 |
| Sodium/potassium-transporting ATPase subunit alpha-1 | P05023 | 22 | 32 |
| Stress-70 protein, mitochondrial | B7Z4V2 | 8 | 11 |
| Suprabasin | Q6UWP8 | 20 | 4 |
| Thioredoxin | P10599 | 16 | 2 |
| Thioredoxin-dependent peroxide reductase, mitochondrial | P30048 | 35 | 32 |
| Thymidine phosphorylase | P19971 | 18 | 0 |
| Transferrin receptor | A8K6Q8 | 5 | 11 |
| Transitional endoplasmic reticulum ATPase | P55072 | 12 | 19 |
| Truncated profilaggrin | I0B0K8 | 17 | 6 |
| Tubulin alpha-1C chain | F5H5D3 | 18 | 0 |
| UDP-glucose ceramide glucosyltransferase-like 1 | A8KAK1 | 7 | 15 |
| Very-long-chain (3R)-3-hydroxyacyl-CoA dehydratase 3 | H3BPZ1 | 6 | 11 |
| Voltage-dependent anion channel 2, isoform CRA_a | A0A024QZN9 | 15 | 17 |
| Zinc-alpha-2-glycoprotein | P25311 | 13 | 12 |

The proteins with either spectral count >10 are listed. BiP and Calnexin are indicated in red. Calreticulin is indicated in blue.
