# Supplementary material for: N-glycosylation in the protease domain of trypsin-like serine proteases mediates calnexin-assisted protein folding
Source: eLife. 2018 Jun 11;7:e35672. doi: 10.7554/eLife.35672 (PMC6021170; doi:10.7554/eLife.35672)
Supplement: Supplementary file 2. [file elife-35672-supp2.docx]

Supplementary File 2. Differentially presented proteins in proteomic analysis.

| protein | UniProt | spectral count | | ratio | |
| --- | --- | --- | --- | --- | --- |
|  | accession | WT | N1022Q | WT/  N1022Q | N1022Q/  WT |
| 14-3-3 protein sigma | P31947 | 26 | 2 | 13.0 | 0.1 |
| 40S ribosomal protein S3 | P23396 | 7 | 16 | 0.4 | 2.3 |
| 40S ribosomal protein SA | A0A024R2P0 | 18 | 7 | 2.6 | 0.4 |
| Alpha-enolase | P06733 | 22 | 5 | 4.4 | 0.2 |
| Arginase-1 | P05089 | 27 | 9 | 3.0 | 0.3 |
| BiP | P11021 | 48 | 94 | 0.5 | 2.0 |
| Bleomycin hydrolase | Q13867 | 15 | 1 | 15.0 | 0.1 |
| Calmodulin-like protein 5 | Q9NZT1 | 39 | 14 | 2.8 | 0.4 |
| Calnexin | P27824 | 13 | 27 | 0.5 | 2.1 |
| Catalase | P04040 | 12 | 3 | 4.0 | 0.3 |
| Chromosome 7 open reading frame 24 | A0A090N7V5 | 15 | 3 | 5.0 | 0.2 |
| Class IVb beta tubulin | Q8IWP6 | 21 | 4 | 5.3 | 0.2 |
| Cystatin-A | C9J0E4 | 13 | 4 | 3.3 | 0.3 |
| Delta-1-pyrroline-5-carboxylate synthase | P54886 | 6 | 23 | 0.3 | 3.8 |
| Elongation factor 1-alpha 1 | P68104 | 29 | 12 | 2.4 | 0.4 |
| Elongation factor 2 | P13639 | 28 | 2 | 14.0 | 0.1 |
| Epididymis luminal protein 4 | D0PNI1 | 20 | 6 | 3.3 | 0.3 |
| Fatty acid-binding protein, epidermal | Q01469 | 45 | 9 | 5.0 | 0.2 |
| Filaggrin-2 | Q5D862 | 70 | 28 | 2.5 | 0.4 |
| Galectin-7 | P47929 | 27 | 0 | +++ | 0.0 |
| Glyceraldehyde-3-phosphate dehydrogenase | P04406 | 80 | 38 | 2.1 | 0.5 |
| Heat shock protein 70 1B | A0A0G2JIW1 | 33 | 13 | 2.5 | 0.4 |
| Heat shock protein 90 beta-1 | P14625 | 52 | 102 | 0.5 | 2.0 |
| Heat shock protein beta-1 | P04792 | 30 | 3 | 10.0 | 0.1 |
| Histone H2B type 1-J | P06899 | 11 | 1 | 11.0 | 0.1 |
| Histone H4 | P62805 | 27 | 9 | 3.0 | 0.3 |
| Insulin-degrading enzyme | P14735 | 11 | 0 | +++ | 0.0 |
| Involucrin | B4DU44 | 11 | 0 | +++ | 0.0 |
| Isoform 2 of Serpin B12 | Q96P63-2 | 15 | 2 | 7.5 | 0.1 |
| L-lactate dehydrogenase A chain | P00338 | 34 | 8 | 4.3 | 0.2 |
| Neutral alpha-glucosidase AB | B4DJ30 | 19 | 43 | 0.4 | 2.3 |
| Peroxiredoxin-1 | Q06830 | 34 | 17 | 2.0 | 0.5 |
| Prelamin-A/C | P02545 | 58 | 4 | 14.5 | 0.1 |
| Protein LOC100653049 | A0A140TA62 | 26 | 0 | +++ | 0.0 |
| Protein POF1B | Q8WVV4 | 21 | 3 | 7.0 | 0.1 |
| Protein S100 | B2R4M6 | 16 | 0 | +++ | 0.0 |
| Protein-glutamine gamma-glutamyltransferase E | Q08188 | 33 | 3 | 11.0 | 0.1 |
| Pyruvate kinase PKM | P14618 | 47 | 13 | 3.6 | 0.3 |
| Serpin B3 | P29508 | 19 | 2 | 9.5 | 0.1 |
| Suprabasin | Q6UWP8 | 20 | 4 | 5.0 | 0.2 |
| Thioredoxin | P10599 | 16 | 2 | 8.0 | 0.1 |
| Thymidine phosphorylase | P19971 | 18 | 0 | +++ | 0.0 |
| Transferrin receptor | A8K6Q8 | 5 | 11 | 0.5 | 2.2 |
| Truncated profilaggrin | I0B0K8 | 17 | 6 | 2.8 | 0.4 |
| Tubulin alpha-1C chain | F5H5D3 | 18 | 0 | +++ | 0.0 |
| UDP-glucose ceramide glucosyltransferase-like 1 | A8KAK1 | 7 | 15 | 0.5 | 2.1 |

The proteins with a ratio of ≥2-fold are listed. +++ indicates the spectral count for WT or N1022Q is 0. ER chaperone proteins are indicated in red.
