# Supplementary material for: N-glycosylation in the protease domain of trypsin-like serine proteases mediates calnexin-assisted protein folding
Source: eLife. 2018 Jun 11;7:e35672. doi: 10.7554/eLife.35672 (PMC6021170; doi:10.7554/eLife.35672)
Supplement: Supplementary file 3. [file elife-35672-supp3.docx]

Supplementary File 3. Information of the DNA inserts in the expression plasmids

| plasmid | DNA insert | NCBI reference sequence | amino acids |
| --- | --- | --- | --- |
|  | (bp of cDNA) |  |  |
| WT | 1-3126 | NM_006587.3 | 1-1042 |
| sWT | 370-3126 | NM_006587.3 | 124-1042 |
| CorinEK4N | 1-2403 | NM_006587.3 | 1-801 |
|  | 2353-3057 | NM_002772.2 | 785-1019 |
| EK-WT | 1-3057 | NM_002772.2 | 1-1019 |
| PT-WT | 1-1866 | NM_000506.4 | 1-622 |
